# Parallelized disruption of prokaryotic and eukaryotic cells via miniaturized and automated bead mill

**DOI:** 10.1002/elsc.202000002

**Published:** 2020-05-06

**Authors:** Roman P. Jansen, Moritz Fabian Müller, Sophie Edith Schröter, Jannick Kappelmann, Bianca Klein, Marco Oldiges, Stephan Noack

**Affiliations:** ^1^ IBG‐1: Biotechnology Forschungszentrum Jülich GmbH Jülich Germany; ^2^ Institute of Biotechnology RWTH Aachen University Aachen Germany; ^3^ Bioeconomy Science Center (BioSC) Forschungszentrum Jülich GmbH Jülich Germany

**Keywords:** bead mill, *C. glutamicum*, cell disruption, Gram‐positive bacteria, proteomics

## Abstract

The application of integrated microbioreactor systems is rapidly becoming of more interest to accelerate strain characterization and bioprocess development. However, available high‐throughput screening capabilities are often limited to target extracellular compounds only. Consequently, there is a great demand for automated technologies allowing for miniaturized and parallel cell disruption providing access to intracellular measurements. In this study, a fully automated bead mill workflow was developed and validated for four different industrial platform organisms: *Escherichia coli*, *Corynebacterium glutamicum*, *Saccharomyces cerevisiae*, and *Aspergillus niger*. The workflow enables up to 48 parallel cell disruptions in microtiter plates and is applicable at‐line to running lab‐scale cultivations. The resulting cell extracts form the basis for quantitative omics studies where no rapid metabolic quenching is required (e.g., genomics and proteomics).

## 1 INTRODUCTION

In recent years accelerated bioprocess development has been realized via miniaturization, automation and parallelization. Increased cultivation and screening throughput have been enabled through the integration of microbioreactor cultivation systems into various robotic platforms [1]. However, most often the screening capabilities are limited to extracellular compounds or fluorescence‐based assays [2, 3, 4]. In order to quantitatively access intracellular target molecules, the cell membrane has to be disrupted releasing cytoplasmic proteins and small intermediates.

Traditionally, cell disruption technologies are divided into two major groups: mechanical and non‐mechanical [5]. Automation of mechanical approaches such as bead mill, French press, or ultrasonicators in a miniaturized and parallelized manner is quite difficult [6]. In a recent study, high‐throughput cell disruption at small‐scale was shown for *Escherichia coli*, but, the method is limited to stand alone applications including a few manual handling steps only [7]. Therefore, only enzymatic or chemical cell disruption methods have been integrated in a fully automated manner on robotic platforms to date. In particular, enzymatic lysis with Lysozyme has been reported to work well for cell disruption of Gram‐negative bacteria such as *E. coli* when being performed with liquid handling systems [8, 9, 10]. However, this strategy becomes insufficient for cell disruption of more robust organisms such as Gram‐positive *Corynebacterium glutamicum*. The addition of harsher detergents is possible, but there is a risk of negatively affecting the amount and activity of any target molecule. If activity is not a factor, e.g., in untargeted proteomics, chemical treatment resulting in denatured proteins is an alternative [11]. Nevertheless, this strategy is currently limited to Gram‐negative bacteria and fungi.

**FIGURE 1:**
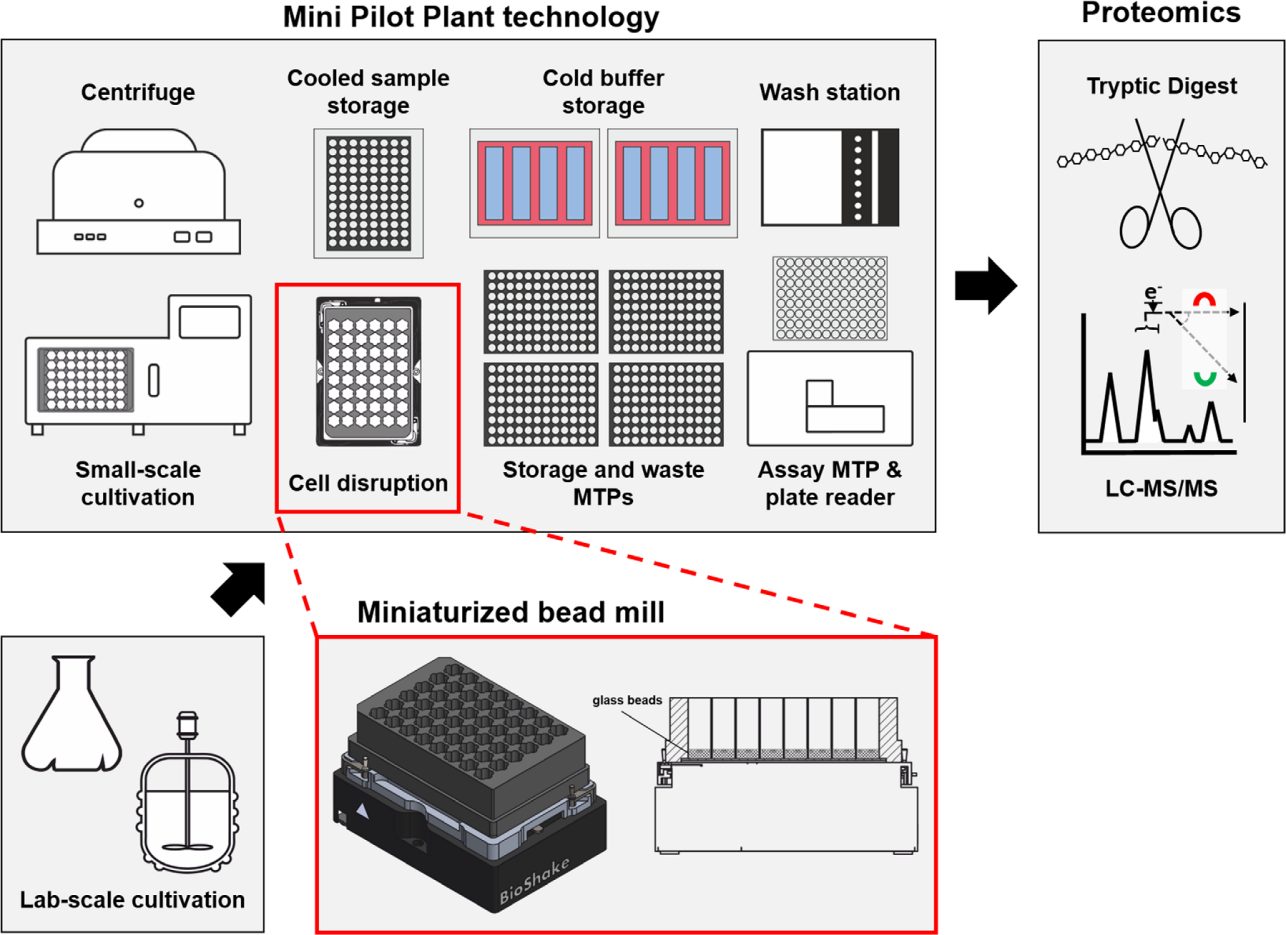
**Principle of the miniaturized bead mill approach applicable for proteomics**. Sample material can be introduced from cultivation experiments performed at various scales including standard lab‐scale devices or small‐scale microbioreactors. Then cells are transferred into a FlowerPlate fixed on a BioShake thermal shaker (side‐view with 500 µL of Ø 0.5 mm glass beads added). The crude cell extract can then be used in a standardized proteomics workflow. In particular, the Mini Pilot Plant technology expanded in this way enables highly automated proteomics experiments

In this study, a miniaturized bead mill workflow was developed, allowing 48 fully automated and parallel cell disruptions for Gram‐negative and Gram‐positive bacteria as well as fungi. The workflow is applicable at‐line to running laboratory‐scale cultivations and provides crude cell extracts for comparative proteome analysis (Figure 1).

## 2 MATERIALS AND METHODS

### 2.1 Cell disruption protocols

All automated cell disruptions were conducted on a robotic platform as previously described by Unthan et al. [12]. After cultivation, 400 µL of cell suspension were transferred via liquid handling system into 1 mL deep well plates and centrifuged at 5000 rpm for 10 min. The culture supernatant was removed and the cell pellets were resuspended with 400 µL 50 mM KP_i_ buffer (pH 7) containing a cOmplete™ EDTA‐free protease inhibitor cocktail (Merck, Darmstadt, Germany), which was stored at 4°C on the robotic deck. A total of 250 µL of the washed cells were then transferred into the prepared FlowerPlate fixed on a BioShake thermoshaker containing 500 µL of Ø 0.5 mm glass beads. The cell disruption was performed at room temperature for 2 min at 3000 rpm. Six cycles of shaking were conducted with 2‐min breaks in between to avoid overheating. After the last cycle, 500 µL of 0.1 mM KP_i_ buffer (pH 7) were added into each well. After a final mixing step for 30 s at 500 rpm, 300 µL of diluted crude cell extract were sampled from the FlowerPlate and transferred into a fresh 96 deep well microtiter plate. The crude cell extract was centrifuged at 5000 rpm for 10 min to remove cell debris. Afterward 250 µL of the supernatant was transferred into a new deep well microtiter plate and stored at 4°C until further use.

The manual bead mill workflow was adapted from Voges et al. [13, 14]. Cells were centrifuged at 4°C for 10 min at 5000 rpm, the supernatant removed, and the cells resuspended in lysis buffer (50 mM KP_i_ buffer (pH 7)) supplemented with a complete protease inhibitor cocktail. The protocol was completely conducted in a 4°C room to reduce possible protein degradation. 500 µL of washed cell suspension together with 500 µL of glass beads with a diameter of Ø 0.1 mm and two Ø 1 mm glass beads were transferred into a 2 mL reaction tube. Via a Retsch bead mill the cells were ruptured mechanically in three cycles at maximum velocity for 2 min. In between each cycle an incubation of at least 60 s on ice was performed to prevent overheating. Afterward, the cell debris was removed through centrifugation at 4°C for 30 min at 16 000 × *g*.

The bead mill cell disruption process utilizing the commercial Precellys System was adapted from Unrean et al. [15]. Cells were centrifuged at 4°C for 10 min at 5000 rpm, the supernatant removed, and the cells resuspended in lysis buffer (50 mM KP_i_ buffer (pH 7)) supplemented with a complete protease inhibitor cocktail. The cell suspensions were then disrupted in a Precellys System (Bertin Instrumentes, Montigny‐le‐Bretonneux, France) with Ø 0.1 mm glass beads in three cycles of each 30 s at maximum frequency. The cell debris was removed with a second centrifugation step at 4°C for 30 min at 16 000 x *g*.

**PRACTICAL APPLICATION**

Miniaturized and automated cell disruption becomes a valuable tool for integrated microbioreactor platforms. Through easy implementation, it enables efficient and parallelized cell disruption for both prokaryotic and eukaryotic cells. Finally, intracellular targets can be accessed for process characterization and optimization, allowing better quantitative phenotyping.

### 2.2 Strains, media, and cultivation conditions

*E. coli* BL21 with a pRSET A plasmid coding for a his‐tagged GFP was cultivated in auto‐induction medium as described by Studier [16]. The complex medium contains per 1 L of deionized water: 12 g peptone, 24 g yeast extract, 0.5 g d‐glucose, 2 g lactose, 90 mL of a 1 M KP_i_ buffer (pH 7), and 5 mL glycerine. A total of 50 mL of auto‐induction medium were inoculated with cryo‐preserved cultures to an OD_6oo_ 0.1 and incubated at 30°C in a 500 mL shake flask with baffles at 300 rpm for 24 h. The gene sequence for the recombinant protein is listed in the supplementary material.

*C. glutamicum* WT::*lacZ* strain as described by Krumbach et al. [17] was cultivated in defined CGXII medium as described by Keilhauer [18]. The medium consists per liter of deionized water of: 20 g d‐glucose, 41.852 g MOPS, 20 g (NH_4_)SO_4_, 5 g (NH_2_)CO, 1 g K_2_HPO_4_, 1 g KH_2_PO_4_, 13.25 mg CaCl_2_·2 H_2_O, 0.25 g MgSO_4_·7 H_2_O, 10 mg FeSO_4_·7 H_2_O, 10 mg MnSO_4_·4 H_2_O, 0.313 mg CuSO_4_·5 H_2_O, 0.02 mg NiCl_2_·6 H_2_O, 1 mg ZnSO_4_·7 H_2_O, 0.2 mg biotin, 30 mg protocatechuic acid. 50 mL of CGXII medium were inoculated with cryo‐preserved cultures to an OD_6oo_ 0.1 and incubated at 30°C in a 500 mL shake flask with baffles at 300 rpm for 24 h.

*Saccharomyces cerevisiae* wild‐type was cultivated in complex YM medium consisting of per liter of deionized water: 10 g d‐glucose, 3 g malt extract, 5 g peptone, and 3 g yeast extract. Fifty milliliters of YM medium were inoculated with cryo‐preserved cultures to an OD_6oo_ 0.1 and incubated at 30°C in a 500 mL shake flask with baffles at 300 rpm for 24 h.

*Aspergillus niger anip7‐gfp2* was cultivated in defined Vogel medium [19, 20]. The medium contains per liter of deionized water: 10 g d‐xylose, 5 mg C_6_H_8_O_7_·H_2_O, 5 mg ZnSO_4_·7 H_2_O, 1 mg Fe(NH_4_)_2_ (SO_4_)_2_·6 H_2_O, 0.16 mg CuSO_4_, 0.5 mg MnCl_2_·H_2_O, 0.05 mg H_3_BO_3_, 0.037 MnSO_4_·H_2_O, 0.05 mg Na_2_MoO_4_·2H_2_O, 6.6 g (NH_4_)SO_4_, 2.5 g KH_2_PO_4_, 0.2 g MgSO_4_·7 H_2_O and 0.1 g CaCl_2_·2 H_2_O. The medium was inoculated with 10^5^ spores/mL and cultivated in a FlowerPlate at 37°C and 1300 rpm for 48 h in a BioLector with an initial filling volume of 1 mL and an increased humidity above 85%.

### 2.3 Analytics

The ß‐galactosidase activity was determined with an adapted protocol based on a published Instruction Manual for the ß‐Galactosidase Assay kit from Agilent. The assay is based on the hydrolysis of the colorless *O*‐nitrophenyl‐β‐d‐galactopyranosid by ß‐galactosidase to d‐galactose and *O*‐nitrophenol, which has a yellow color that can be detected at 420 nm. For the microtiter plate assay 0.1 mg/mL ONPG were dissolved in 0.1 M KP_i_ buffer (pH 7). A total of 175 µL of the substrate solution were then added to 25 µL of the crude cell extract and the absorbance was measured at 420 nm for 10 min. The enzyme activity was calculated and used as a measure of cell rupture efficiency.

Cell dry weight was determined gravimetrically with pre‐weighed and dried 2 mL reaction tubes. One milliliter of culture suspension was transferred into a labelled reaction tube and centrifuged for 5 min at 5000 × *g*. The cell pellet was washed with 0.9% (w/v) NaCl and centrifuged again. The supernatant was removed and the pellet dried at 80°C until a constant weight was reached.

## 3 RESULTS AND DISCUSSION

### 3.1 Development and optimization of miniaturized bead mill approach

Combining specialized microtiter plates (FlowerPlates^®^, m2p‐labs) with an integrated thermoshaker, centrifuge, and plate reader on a robotic liquid handling system enables automated cell disruption (Figure 1). Through the addition of small glass beads, the principle of a standard bead mill can be imitated in a parallelized and miniaturized manner. The established protocol allows the processing of 48 biomass containing samples in parallel, which might be obtained from transient sampling of one culture or single sampling of certain replicate cultures.

First, cells are transferred by the liquid handling system, centrifuged and resuspended in 0.9% (w/v) NaCl solution to remove culture supernatants. Second, the resuspended cells are transferred into the prepared FlowerPlate fixed on the BioShake (Figure 1). Third, cell disruption is performed with cycles of 2 min shaking at 3000 rpm with 2 min breaks in between to minimize possible heat production with subsequent protein denaturation [21]. Finally, the suspension is centrifuged to remove cell debris and the total soluble protein supernatant is stored at 4°C until further use.

In order to identify optimal cell disruption conditions, parameters such as bead type, bead size, duration, and bead to cell suspension ratio were tested (Table 1). To focus on technical reproducibility of the cell disruption step, equivalent biomass containing samples from shake flask cultures were prepared for each optimization criteria and adjusted to OD_600_ 10. Initial optimization of bead type, bead size, and cycle number was conducted with *E. coli* whereas bead to cell ratio was investigated with *C. glutamicum* as a much more challenging and robust organism (Table 1).

**TABLE 1:** **Development and optimization of miniaturized bead mill approach**. The conditions highlighted in **grey** were selected as best setting

Parameter (tested organism)	Condition	Soluble protein concentration (mg/L)
Bead type (*E. coli*)	Zirconium beads	0.57 ± 0.02
	**Glass beads**	**0.98 ± 0.02**
Bead size (*E. coli*)	without beads	0.17 ± 0.01
	Ø 0.1 mm	1.17 ± 0.02
	Ø 0.3 mm	1.22 ± 0.05
	**Ø 0.5 mm**	**1.33 ± 0.04**
	Ø 0.7 mm	0.97 ± 0.06
Cycle number (*E. coli*)	3	1.20 ± 0.05
	4	1.21 ± 0.07
	5	1.35 ± 0.11
	**6**	**1.41 ± 0.08**
	7	1.45 ± 0.16
	8	1.45 ± 0.10
Cell to bead ratio in µL (*C. glutamicum*)	500:500	0.23 ± 0.02
	**250:500**	**0.62 ± 0.02**
	150:500	0.85 ± 0.09
	125:500	0.92 ± 0.09

Direct comparison of zirconium and glass beads of two sizes showed that glass beads provided better performances and were selected for further optimization. The investigation of bead size diameter from 0.1 to 0.7 mm resulted in the selection of 0.5 mm beads showing the highest protein concentration in the crude cell extracts. By raising the number of the 2‐min cell disruption cycles from three up to seven the protein concentrations could be consecutively increased until almost saturation after six cycles. Moreover, reducing the cell to bead ratio from 500:500 to 125:500 proved to be beneficial as well. Finally, to avoid potential overheating by too many cycles as well as blockage of the liquid handling system at too low cell to bead ratio, the cycle number was set to six and the cell to bead ratio to 250:500 µL, respectively.

### 3.2 Validation at the level of total soluble protein and target enzyme activity

**FIGURE 2:**
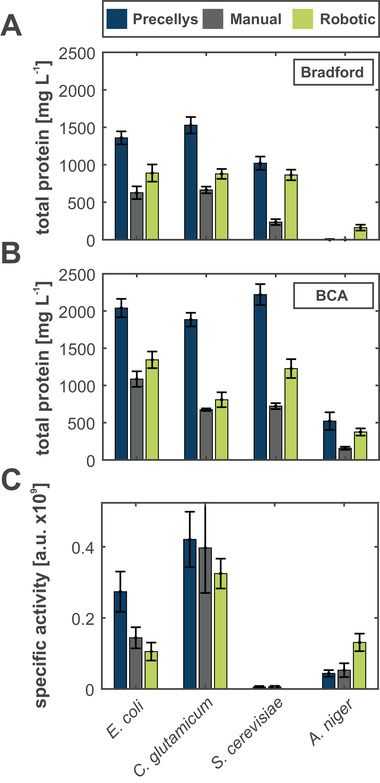
**Validation of miniaturized bead mill approach at the level of total protein and target enzyme activity**. Total protein concentrations were analyzed with BCA [22] and Bradford [23] assay, respectively. Activity of ß‐galactosidase (native in *E. coli* and *A. niger*, recombinant in *C. glutamicum*, not present in *S. cerevisiae*) was determined with a kinetic assay based on the hydrolysis of *O*‐nitrophenyl‐β‐d‐galactopyranosid to galactose and *O*‐nitrophenol. Specific values are related to the total amount of protein in each sample

For a first validation of the miniaturized (robotic) bead mill approach, it was compared to a manual bead mill method using conventional Eppendorf tubes [13] and a commercial system, the Homogenisator from Precellys (Bertin Instrumentes) [15]. Cell lysis performance of all three approaches based on total soluble protein as well as activity of ß‐galactosidase as selected target enzyme (expressed in auto‐induction medium) was compared for four different industrial platform organisms, namely *E. coli*, *C. glutamicum*, *S. cerevisiae*, and *A. niger* (Figure 2).

As a result, the performance ranking for the three cell disruption methods is nearly identical for both protein determination assays, *i.e*., Bradford and BCA, and all tested organisms (Figure 2A and B). Noteworthy, the BCA assay consistently resulted in higher protein concentrations, which might be explained by its higher sensitivity in contrast to the Lowry method. Please note that the commercial bead mill system outperforms the manual and the robotic approach. This is likely due to an additional freezing step in the protocol, which might facilitate breakage of cell wall and membrane structures. The protein concentrations of 2039 ± 123 mg/L and 1884 ± 91 mg/L for *E. coli* and *C. glutamicum*, respectively, are close to the maximal experimental yields based on literature [24, 25] for an initial biomass of OD_600_ 10, which is approximately 3.5 g/L cell dry weight. Most importantly, the robotic workflow consistently results in higher total protein concentrations compared to the manual cell lysis protocol, making it a suitable alternative. More specifically, 134 ± 11 µg and 81 ± 10 µg of soluble protein per OD_600_ were detected for *E. coli* and *C. glutamicum*, respectively. These results match very well with recent literature data where approx. 90 µg protein per OD_600_ was obtained from *E. coli* cells, treated by an automated chloroform/methanol cell lysis procedure [11].

Finally, specific ß‐galactosidase activity was successfully determined in *E. coli*, *C. glutamicum*, and *A. niger* following all three cell lysis methods (Figure 2C). While the Precellys system performed best for *E. coli* and *C. glutamicum*, the robotic bead mill approach was comparable to the manual cell lysis protocol and even resulted in the highest activity for *A. niger*. This proves that the proposed miniaturized bead mill approach enables a gentle cell disruption, providing comparable activity data and gives rise to the conclusion that the obtained proteins are still active and not denatured.

### 3.3 Validation at the level of single cytosolic proteins

For a second validation of the robotic bead mill approach, it was applied in a comparative proteome analysis with the Precellys system as reference method. From a single *C. glutamicum* shake flask cultivation in defined CGXII medium 12 technical replicate samples were taken and processed individually for each cell disruption technology leading to a total sample number of 50. Each of the samples was then injected as a fivefold analytical replicate for LC–MS/MS analysis.

As a result, a comparably high number of 1148 individual proteins were detected with both workflows, proving that the miniaturized bead mill approach is suitable for comparative proteomics studies. Strikingly, only three out of all identified proteins (≈ 0.3%) showed a slight but significant change between both methods (Supporting Information Figure S1). From these three only the protein with accession number CAF21289 has a clear functional annotation as transcriptional regulator LacI [26]. LacI is involved in the regulation of lactose utilization, but, why it was found upregulated when applying the bead mill approach needs further investigation.

## 4 CONCLUDING REMARKS

The presented miniaturized bead mill approach enables efficient and reproducible cell disruption for up to 48 samples in an automated manner. It is applicable to different industrial platform organisms, including Gram‐positive bacteria such as *C. glutamicum* as well as filamentous fungi such as *A. niger*. Quantification of total soluble protein in crude cell extracts, determination of enzyme activities with standardized enzyme assays or comparative proteomics studies can now be initiated in a much faster way. Moreover, in combination with the Mini Pilot Plant technology [12], this workflow can be applied for quantitative microbial phenotyping with an increased throughput for intracellular studies where no rapid metabolic quenching is required (e.g., genomics and proteomics).

## CONFLICT OF INTEREST

The authors declare no conflict of interest.

## AUTHOR CONTRIBUTIONS

RJ and MFM planned, supervised and analysed all experiments. SES conducted most of the miniaturized cell disruptions in order to find optimal conditions. BK and JK conducted all proteomics experiments and helped with their analysis. MO proof read all versions of the manuscript and helped finalizing it. SN helped co‐writing the manuscript and supervised the entire project. All authors read and approved the final manuscript.

## Supporting information

Supporting Information
