# Supplementary material for: Parallelized disruption of prokaryotic and eukaryotic cells via miniaturized and automated bead mill
Source: Eng Life Sci. 2020 May 6;20(8):350–6. doi: 10.1002/elsc.202000002 (PMC7401235; doi:10.1002/elsc.202000002)
Supplement: Supplementary file 1 — Supporting Information [file ELSC-20-350-s001.pdf]

Supplementary Material

Parallelized disruption of prokaryotic and eukaryotic cells via miniaturized bead mill

Roman P. Jansen[1,2,#], Moritz Fabian Müller[1,#], Sophie Edith Schröter[1], Jannick Kappelmann[1], Bianca Klein[1], Marco Oldiges[1,2,3], Stephan Noack[1,3,*]

The gene sequence for the *E. coli BL21* with a pRSET A plasmid coding for a his-tagged GFP used in this study is as follows:

ATGGTGAGCAAGGGCGAGGAGCTGTTCACCGGGGTGGTGCCCATCCTGGTCGAGCTGGACGGCGACGTAAAC
GGCCACAAGTTCAGCGTGTCCGGCGAGGGCGAGGGCGATGCCACCTACGGCAAGCTGACCCTGAAGTTCATCT
GCACCACCGGCAAGCTGCCCGTGCCCTGGCCCACCCTCGTGACCACCCTGACCTACGGCGTGCAGTGCTTCAGC
CGCTACCCCGACCACATGAAGCAGCACGACTTCTTCAAGTCCGCCATGCCCGAAGGCTACGTCCAGGAGCGCA
CCATCTTCTTCAAGGACGACGGCAACTACAAGACCCGCGCCGAGGTGAAGTTCGAGGGCGACACCCTGGTGAA
CCGCATCGAGCTGAAGGGCATCGACTTCAAGGAGGACGGCAACATCCTGGGGCACAAGCTGGAGTACAACTA
CAACAGCCACAACGTCTATATCATGGCCGACAAGCAGAAGAACGGCATCAAGGTGAACTTCAAGATCCGCCAC
AACATCGAGGACGGCAGCGTGCAGCTCGCCGACCACTACCAGCAGAACACCCCCATCGGCGACGGCCCCGTGC
TGCTGCCCGACAACCACTACCTGAGCACCCAGTCCGCCCTGAGCAAAGACCCCAACGAGAAGCGCGATCACAT
GGTCCTGCTGGAGTTCGTGACCGCCGCCGGGATCACTCTCGGCATGGACGAGCTGTACAAGCTCGAGCACCAC
CACCACCACCACTGA

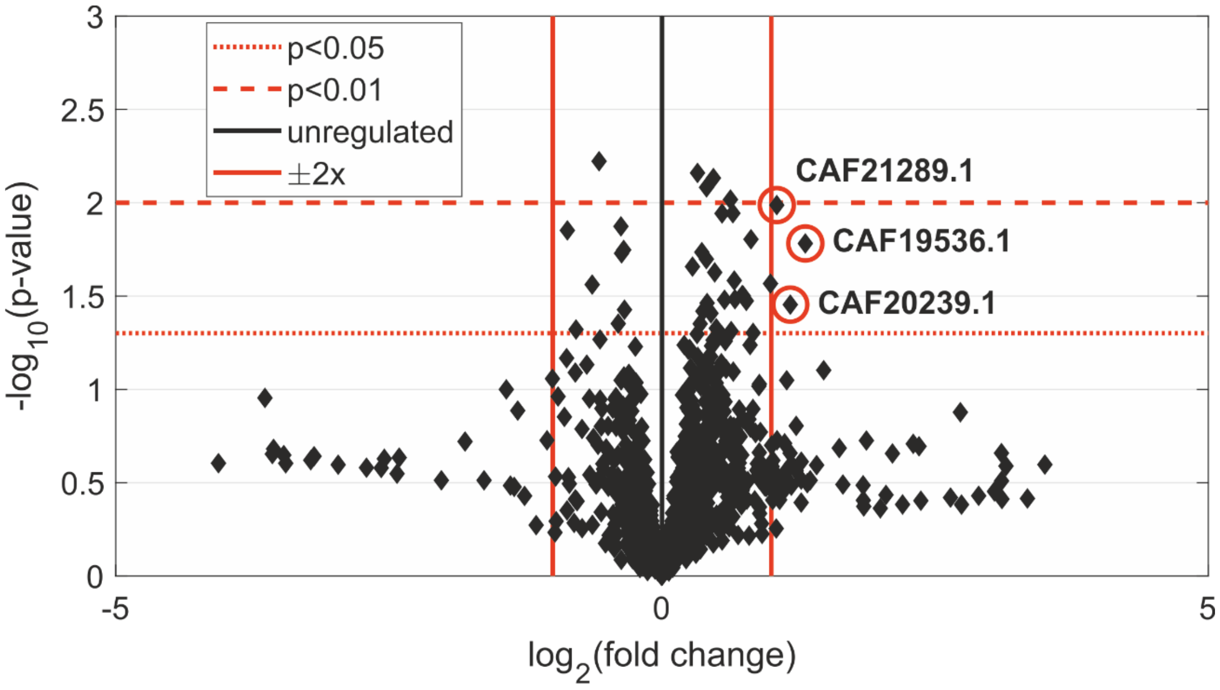

Supplementary Figure 1: Volcano Plot of protein fold changes when applying the miniaturized bead mill approach in comparison to the commercial Precellys system. For the determination of significantly changed proteins, p-values of 0.01 and 0.5 were set as threshold
